# Moderate thinning enhances soil carbon-nitrogen cycling and microbial diversity in degraded mixed forests

**DOI:** 10.3389/fmicb.2025.1652531

**Published:** 2025-10-02

**Authors:** Yu Wang, Baoshan Zhang, Xue Yang, Fei Xu

**Affiliations:** ^1^College of Environmental Science and Engineering, China West Normal University, Nanchong, China; ^2^Chinese Research Academy of Environmental Sciences, Beijing, China

**Keywords:** soil carbon-nitrogen dynamics, microbial community structure, thinning intensities, degraded forest, ecosystem function

## Abstract

**Introduction:**

Soil carbon and nitrogen components play a crucial role in maintaining ecosystem functions and regulating global climate change in forest ecosystems. Thinning is an important forest management measure that significantly affects forest structure and biodiversity. However, the specific impacts of varying thinning intensities on soil carbon-nitrogen dynamics and microbial community structure remain unclear, warranting further investigation.

**Methods:**

In this study, we applied a gradient of thinning intensities (10–35%) in a degraded mixed forest, combining field sampling, soil physicochemical measurements, and high-throughput sequencing to assess changes in soil carbon-nitrogen components and microbial communities. We used Mantel tests to quantify correlations between soil environmental factors and microbial community composition, variance partitioning analysis (VPA) to determine the relative contributions of carbon and nitrogen variables, and Boruta-based random forest modeling to identify the most important predictors of microbial variation. Mixed-effects models (controlling for elevation, slope, SI_70_) were used to test thinning effects on nitrogen fractions and C/N.

**Results:**

Baseline (pre-treatment) soils exhibited high bacterial α-diversity but lower and more variable fungal diversity, with intermediate SOC and TN levels across plots. In contrast, fungal communities showed a simpler response, primarily influenced by the C/N ratio, dissolved organic carbon (DOC), and total nitrogen (TN). Mixed-effects models (controlling for elevation, slope, SI_70_) showed independent thinning effects on DON, MBN, NH_4_^+^-N, TN and C/N, but not on AN or NO_3_^−^-N. VPA showed that, for bacteria, carbon explained 26.86%, nitrogen 35.50%, and their interaction 29.04%; for fungi, 19.92, 38.68, and 34.87%, respectively—indicating nitrogen’s dominant role. The C/N ratio, TN, and NO_3_^−^-N had the highest explanatory power for thinning intensity, suggesting that nitrogen dynamics play a more significant role.

**Discussion:**

The findings of this study improve the understanding of how forest management practices influence soil carbon-nitrogen, providing scientific evidence for the precise regulation of forest ecosystem functions and services, with significant implications for ecological management and climate regulation.

## 1 Introduction

Soil carbon-nitrogen dynamics are essential for global ecosystem functioning and climate change mitigation, as they are closely tied to Earth’s elemental cycles and climate regulation (Liu et al., 2018; Parolari and Porporato, 2016). The structure of soil microbial communities directly influences soil carbon-nitrogen stocks and cycling efficiency by driving processes such as transformation, decomposition, and fixation (Winsome et al., 2017; Yokobe et al., 2018). Existing research has yet to systematically explore how thinning treatments influence forest soil carbon-nitrogen dynamics and microbial community structure, leaving an incomplete understanding of forest soil functions and ecosystem services (Bai et al., 2017; Dang et al., 2018; Xu J. et al., 2025). Thinning treatments are widely used in practice, yet their role in regulating soil carbon-nitrogen dynamics and microbial communities remains poorly understood, highlighting the need for further studies (Gross et al., 2018; Xu et al., 2024b). Therefore, exploring the effects of different thinning intensities on soil carbon-nitrogen dynamics and microbial community structure is crucial for evaluating the effectiveness and ecological consequences of forest management strategies (Lei et al., 2021; Zhang Z. et al., 2023).

Much research has examined the impact of climate factors (e.g., temperature and humidity), land use practices, and disturbance intensity on soil carbon-nitrogen dynamics and microbial community structure (Sun et al., 2020; Tourville et al., 2022). For example, studies on reforestation and forest management have shown that changes in vegetation cover significantly alter soil carbon-nitrogen stocks and turnover rates, as well as microbial community diversity and functional structure (Aerts and Honnay, 2011; Zhang et al., 2025). Long-term monitoring has shown that extreme disturbances, such as overharvesting or fire, significantly reduce soil organic matter content and the abundance of key microbial groups (e.g., nitrogen-fixing bacteria, cellulose-decomposing bacteria), weakening soil nutrient supply capacity (Xu Y. et al., 2025). Further studies suggest that soil carbon-nitrogen dynamics are closely linked to microbial physiological traits, with microbial communities showing variations in carbon and nitrogen substrate utilization efficiency under different ecological conditions (Ren et al., 2024; Wang et al., 2025). Despite these studies providing a solid foundation for understanding forest soil carbon-nitrogen cycling, a systematic exploration of soil carbon-nitrogen indicators and microbial community structure under varying thinning intensities is still lacking, making it difficult to accurately assess thinning’s impact on soil ecological processes.

Thinning is a common and effective management technique in forest management that regulates stand density and ecological factors, such as light, moisture, and nutrients (Song et al., 2024; Xu et al., 2024b; Zhou et al., 2020). This technique provides an ideal platform for studying soil carbon-nitrogen dynamics while maintaining forest health and productivity. Compared to large-scale or uncontrollable disturbance methods, thinning treatments with varying intensities offer greater controllability and flexibility (Xu et al., 2022). Researchers can simulate varying degrees of thinning by adjusting thinning ratios and use multiple indicators (e.g., soil organic carbon, total nitrogen, microbial biomass carbon-nitrogen, enzyme activity) to assess thinning’s effects on the soil environment and microbial communities (Guo et al., 2024; Li et al., 2024). Thinning also creates favorable conditions for studying the coupling between microbial communities and soil carbon-nitrogen dynamics (Kim et al., 2019; Li et al., 2023). Through strict experimental design, including sampling points, periods, and thinning intensities, researchers can reveal how ecological factors influence carbon-nitrogen cycling (Chen et al., 2015; Wu et al., 2019). Thus, a systematic study of soil carbon-nitrogen dynamics and microbial community structure under varying thinning intensities will not only fill existing research gaps but also provide scientific evidence for the precise regulation of forest ecosystem functions and services (Lei et al., 2021; Song et al., 2024).

This study provides the first integrated assessment in mixed-wood forests that couples a volume-defined thinning gradient (0–35%) with joint quantification of soil C-N fractions and high-throughput microbial communities. Here we systematically evaluate how a volume-defined gradient of thinning intensity reshapes soil C-N dynamics and microbial communities in mixed-wood forests-an integrated design not yet reported for this system. We combined plot-level field sampling, soil physicochemical measurements, and high-throughput sequencing to quantify changes in C-N fractions and community structure across 10–35% thinning. Specifically, the study aimed to achieve the following three objectives: (1) to quantitatively assess the impact of different thinning intensities on soil carbon-nitrogen dynamics; (2) to systematically explore the changes in soil microbial community structure and the abundance of key functional microbial groups under varying thinning intensities; and (3) to elucidate the potential coupling relationships between soil carbon-nitrogen dynamics and microbial community characteristics. By clarifying how tailored thinning regimes reshape soil C-N dynamics and microbial functionality, we aim to equip forest managers and policymakers with actionable guidance for restoring degraded stands while safeguarding ecosystem services and climate-change mitigation potential.

## 2 Materials and methods

### 2.1 Overview of the study area

**Figure 1:**
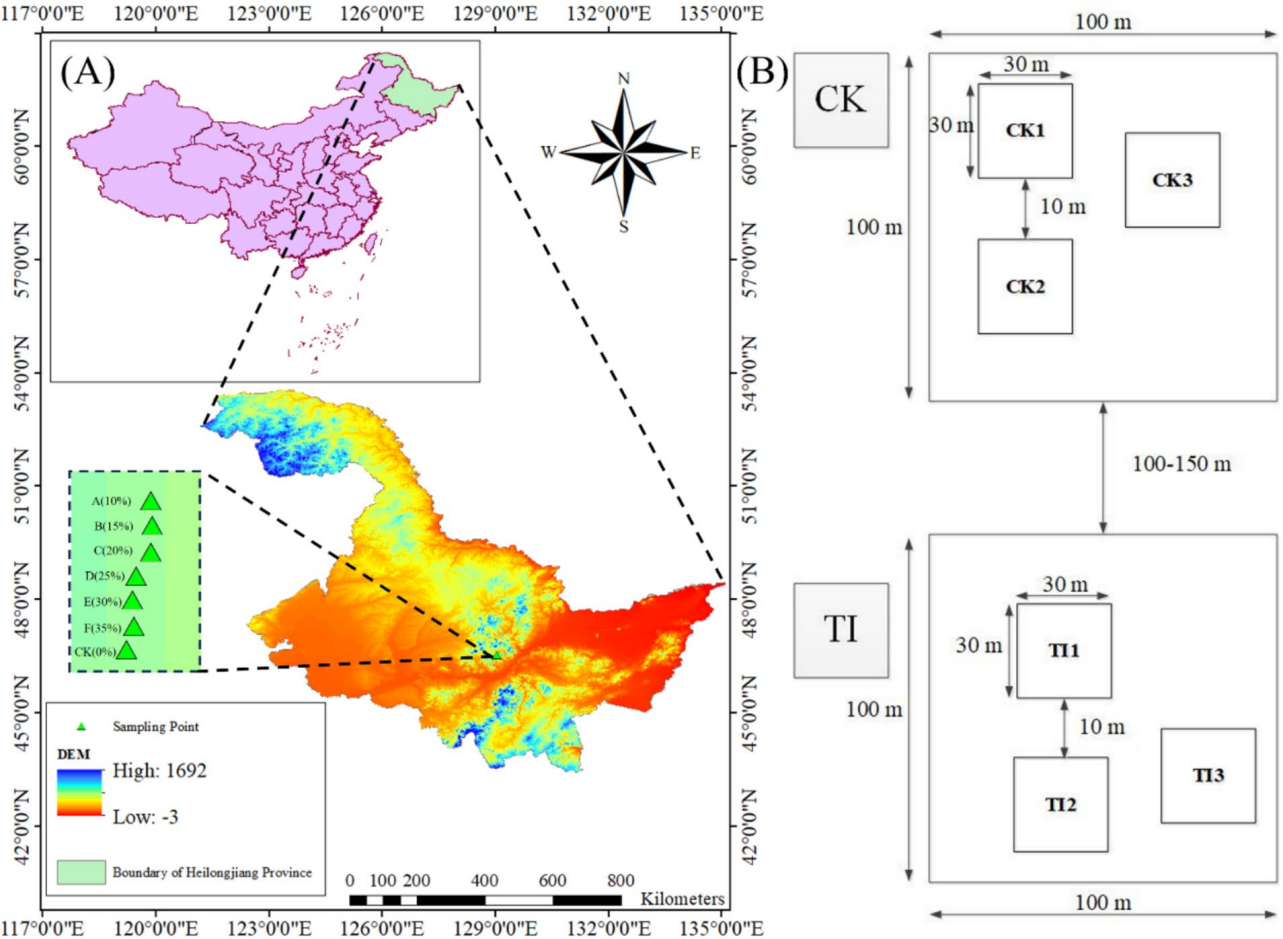
**(A)** Experimental and control plots were established across seven different thinning intensities. **(B)** Plot layout. Each plot is 100 × 100 m and contains three 30 × 30 m subplots (CK1–CK3 or A1–A3, etc.) separated by a 10 m buffer to minimize edge effects; distances among plots are 100–150 m. The same layout applies to all treatment plots A–F.

A forest management experiment was carried out at Dongfanghong Forest Farm in the Daqingshan District, Yichun City, within the Lesser Khingan Mountains of northeastern China (128°37′–129°17′E, 46°50′–47°21′N) (Figure 1A). The region has a continental humid monsoon climate with a mean elevation of ~600 m and annual precipitation of ~660 mm concentrated in July–August. Soils are predominantly dark brown, supporting mixed conifer-broadleaf forests. Prior to thinning, stands averaged ~70 years of age. However, the forest in this area has experienced significant degradation, characterized by low species diversity, disrupted stand structure, and ecosystem fragility (Shi et al., 2022; Zhang B. et al., 2024).

In 2011, seven 100 × 100 m plots were established under comparable stand conditions: an unthinned control (CK) and six thinned plots (A–F). Pre-thinning stand density and pre-thinning merchantable volume (*V*_pre_) were obtained from a tree-by-tree inventory. Thinning followed a low-thinning (from below) prescription, removing suppressed, diseased, dying, or poor-form trees. Thinning intensity was defined by merchantable volume removal at the plot level, by merchantable volume (I_(v)_), calculated as in Equation 1:

(1) $$I_{v}(\%)=\frac{V_{\text{removed}}}{V_{\mathrm{pre}}}\times 100\%$$

where *I*_v_ denotes the thinning intensity by merchantable volume (percent); where *V*_pre_ is the pre-thinning stand merchantable volume per plot (m^3^ ha^−1^) from the tree-by-tree census, and *V*_removed_ is the sum of merchantable volume of all felled trees in that plot (m^3^ ha^−1^), estimated from DBH and height using species-specific volume equations. Target levels were 0, 10, 15, 20, 25, 30, and 35% for CK and A–F, respectively, enforced by tree marking and harvest tallies. For exposition only, we refer to 10–15% as “light,” 20–25% as “moderate,” and 30–35% as “heavy”; statistical models used the exact percentage as a continuous covariate. Immediately after thinning, all thinned plots were gap-filled with *Pinus koraiensis* Siebold & Zucc. and *Picea koraiensis* Nakai under a uniform operational guideline across plots (same stock class and planting window; no fertilization). The guideline specified gap-based planting to restore target stocking using a consistent spacing template and the same handling and planting procedures in A–F. Plot-level planting tallies were not retained; therefore, reforestation is not modeled as a fixed effect, and any residual differences among plots are absorbed by the PlotID random intercept in the mixed-effects models.

Each treatment comprised one 100 × 100 m plot containing three 30 × 30 m subplots separated by a 10-m buffer to minimize edge effects (Figure 1B). Subplots served as subsamples nested within plot and were not treated as independent replicates. Plots were spaced 100 m apart to maintain similar slope, aspect, and forest type. In 2021 (10 years post-treatment), we surveyed species composition, DBH, tree height, and stem density (DBH >5 cm) in all seven plots. Site descriptors (topography and coordinates) are listed in Table 1, and stand metrics before thinning (2011) and at assessment (2021)-with absolute and relative changes-are summarized in Supplementary Table S2 (mean ± SD; three subplots per plot).

**Table 1:** General information of study site.

Plot name	Geographic coordinates		Altitude/m	Aspect	Gradient/(°)	Tree	
	Latitude	Longitude				Average DBH/cm	Average tree height/m
CK (0)	46°52′27.80″	129°05′12.84″	523	Northwest	11	16.0 ± 8.9	15.2 ± 7.1
A (10%)	46°52′28.63″	129°05′08.94″	511	Northwest	11	16.4 ± 9.2	14.5 ± 6.7
B (15%)	46°52′46.12″	129°05′03.00″	459	Northwest	11	14.6 ± 8.4	12.0 ± 4.0
C (20%)	46°52′37.00″	129°05′09.00″	440	Northwest	10	11.8 ± 7.0	10.8 ± 3.7
D (25%)	46°52′40.71″	129°05′09.48″	495	Northwest	10	18.1 ± 8.7	13.1 ± 4.4
E (30%)	46°52′43.00″	129°05′10.00″	460	Northwest	12	18.5 ± 9.3	20.4 ± 9.5
F (35%)	46°52′50.00″	129°05′11.00″	461	Northwest	12	17.8 ± 8.4	13.1 ± 5.1

The values are shown as mean ± standard deviation. Pre- (2011) and assessment-year (2021) stand metrics (density, DBH, height; mean ± SD) and changes are reported in Supplementary Table S2.

### 2.2 Soil sampling

In July 2021, soil was sampled in all seven 100 × 100 m plots. Each plot contained three 30 × 30 m subplots separated by a 10-m buffer. Within each subplot we used a randomized-start five-point layout (four corners + center). After removing the organic horizon, one 0–20 cm mineral-soil core was collected at each point using a 5-cm spiral auger. The five cores were homogenized to one composite per subplot. Thus, each plot contributed three composites, for a study total of *n* = 21 composites (three subplots × seven plots). Composites were passed through a 2-mm sieve to remove coarse fragments and roots; aliquots were kept chilled for physicochemical analyses and stored at −80 °C for DNA.

### 2.3 Analysis of properties and carbon-nitrogen components

Soil pH was measured by shaking a soil: water (1:5 w/v) suspension for 30 min and using a pH meter (Sartorius AG, Goettingen, Germany) (Bao, 2000). Soil temperature (ST) and soil moisture (SM) levels were precisely measured using the LI-8150 system (Lincoln, NE, United States), utilizing an E-type thermocouple in combination with an EC-5 soil moisture probe (Wang and Li, 2025). The soil bulk density is conducted using the ring knife method (Scanlon et al., 2002). Total phosphorus (TP) was measured via colorimetric analysis after wet digestion with H_2_SO_4_ and HClO_4_ (Sommers and Nelson, 1972). Meanwhile, available phosphorus (AP) was evaluated using the molybdenum blue method (Zhang et al., 2015). Total potassium (TK) content was assessed using the Thermo Scientific iCE 3300 Atomic Absorption Spectrometer (Thermo Fisher Scientific, Massachusetts, United States). Utilizing the NH_4_OAc extraction method, we measured the levels of AK in the soil (Xu et al., 2019).

The soil organic carbon (SOC) content was determined using the potassium dichromate sulfuric acid heating method (Li et al., 2021). The easily oxidizable carbon (EOC) content in soil was determined through a potassium permanganate oxidation-colorimetric method (Blair et al., 1995). Dissolved organic carbon (DOC) and dissolved organic nitrogen (DON) content were analyzed using the EuroEA3000 element analyzer (Leeman Company, United States) (Gonet and Debska, 2006). Soil total nitrogen (TN) content was quantified using the Kjeldahl method, while the available nitrogen (AN) content was assessed through the Alkali diffusion method (Tang et al., 2021). Ammonium nitrogen (NH_4_^+^-N) and nitrate nitrogen (NO_3_^−^-N) concentrations were quantified utilizing a 1 M KCl extraction method and an AA3 continuous flow analytical system (AA3, Germany) (Dang et al., 2018). The microbial biomass carbon (MBC) and microbial biomass nitrogen (MBN) were determined using the chloroform fumigation extraction method, and a conversion factor of 0.45 was applied to both measurements (He et al., 2015). C/N ratio was calculated by the ratio of SOC and TN (Lou et al., 2012). Pre-thinning baseline values reported in Supplementary Table S1 were derived from the 2011 pre-treatment sampling (three 30 × 30 m subplots per plot; *n* = 21 composites). Variables include SOC, EOC, DOC, MBC, TN, AN, DON, NH_4_^+^-N, NO_3_^−^-N, MBN, and bacterial/fungal α-diversity indices.

### 2.4 Soil DNA extraction and sequencing

Soil DNA was extracted in triplicate from each sample using the E.Z.N.A. Soil DNA Kit (OMEGA, United States) according to the manufacturer’s protocol (Tzelepis et al., 2017). Briefly, 0.5 g of soil was treated with a detergent buffer, then heated and frozen to remove contaminants. The purified DNA was subsequently eluted in water or a low ionic strength buffer. Duplicate extracts were combined and quantified using a NanoDrop ND-1000 spectrophotometer (Thermo Fisher Scientific). DNA was stored at −80 °C until PCR amplification and sequencing of the 16S rRNA gene and ITS regions.

The V3–V4 region of the 16S rRNA gene was amplified using primers 341F and 806R, Phusion^®^ High-Fidelity Master Mix, 2 μM primers, and approximately 10 ng of DNA. Thermal cycling consisted of 30 cycles of denaturation at 98 °C, annealing at 50 °C, and extension at 72 °C, followed by a final extension step. Similarly, the ITS1 region was amplified using primers ITS3-2024-F and ITS4-2049-R with 35 thermal cycles at appropriate temperatures (Francioli et al., 2021). PCR products were verified using a 2% agarose gel, pooled, and purified with the Qiagen Gel Extraction Kit (Qiagen, Germany).

Sequencing libraries were prepared using the TruSeq^®^ DNA PCR-Free Kit (Illumina, United States) and included index addition. Library quality was assessed using a Qubit^®^ 2.0 Fluorometer and an Agilent Bioanalyzer 2100. Libraries were sequenced on an Illumina NovaSeq platform (250 bp paired-end reads) by Novogene Bioinformatics Technology Co., Ltd. (Beijing, China).

### 2.5 Sequence data processing

The assembly and quality control of paired-end reads involved several steps. First, paired-end reads were assigned to their respective samples using unique barcodes, after which the barcode and primer sequences were removed. Next, the FLASH tool (V1.2.7, http://ccb.jhu.edu/software/FLASH/) was used to merge paired-end reads by aligning overlapping sequences from opposite ends of the same DNA fragment, generating raw tags. To ensure data quality, raw tags underwent stringent filtration based on specific criteria, resulting in high-quality clean tags. This filtration procedure followed the quality control protocol outlined in QIIME (V1.9.1, http://qiime.org/scripts/split_libraries_fastq.html). Additionally, obtained tags were compared against the reference Silva database^1^ using the UCHIME algorithm to identify and eliminate chimeric sequences. Ultimately, the remaining sequences were considered effective tags, ready for further analysis. Sequence analysis was performed using Uparse software (Uparse v7.0.1001, http://drive5.com/uparse/). Sequences with ≥97% similarity were grouped into OTUs, and a representative sequence from each OTU was selected for further annotation. Taxonomic information was assigned to each representative sequence using the Mothur algorithm and the Silva Database. To explore the phylogenetic relationships among OTUs and assess differences in dominant species across sample groups, we used MUSCLE software (Version 3.8.31, http://www.drive5.com/muscle/) for multiple sequence alignment. OTU abundance was standardized using the sample with the lowest sequence count as a reference. Subsequent analyses were performed using this normalized dataset. The 16S rRNA gene sequences were aligned with PyNAST software (Version 1.2) against the “Core Set” data from the GreenGene database for rapid multiple sequence alignment. For the 18S and ITS regions, MUSCLE was used to align sequences against their respective reference sequences to determine the phylogenetic relationships of all OTU representative sequences.

### 2.6 Statistical analysis

Before testing thinning effects, we summarized 0–20 cm baseline soil and microbial metrics (*n* = 21; Supplementary Table S1). We first summarized site-level baseline metrics for C-N fractions and α-diversity (All plots, *n* = 21; Supplementary Table S1). We fitted linear mixed-effects models (ML estimation; Satterthwaite df) with PlotID as a random intercept to test thinning effects while accounting for between-plot differences. Fixed effects included continuous thinning intensity (TI) and z-standardized elevation, slope, and site index at age 70 (SI_70_). For each response (TN, NH_4_^+^-N, NO_3_^−^-N, AN, DON, MBN, C/N), we compared a covariate-only model (M0) with M1 = M0 + TI using AIC and −2 log-likelihood (−2LL). We report Type III tests for fixed effects, parameter estimates, and the intraclass correlation coefficient (ICC = Var [Intercept|Plot]/[Var [Intercept|Plot] + Var [Residual]]) (Supplementary Table S3). Reforestation was not included as a fixed effect due to the absence of plot-level planting tallies; potential plot-to-plot differences associated with reforestation are accommodated by the random plot intercept in the LMMs.

A one-way analysis of variance (ANOVA) was conducted using SPSS 22.0, followed by least significant difference (LSD) multiple comparisons (*p* < 0.05) to evaluate the significant effects of different treatments on soil properties (pH, ST, SM, BD, TP, TK, AP, AK, C/N), carbon components (SOC, EOC, DOC, MBC), and nitrogen components (TN, AN, DON, NH_4_^+^-N, NO_3_^−^-N, MBN). To visualize variations in microbial communities across seven thinning intensities, flower plots were generated using the *VennDiagram* package in R. Phylogenetic trees were constructed using FastTree software^2^ based on the approximate maximum likelihood algorithm. LEfSe analysis (v1.0) was performed to investigate differences in microflora across thinning intensities using the linear discriminant analysis effect size (LEfSe) method (score >3.5) (Segata et al., 2011). This allowed the identification of significant differences in bacterial and fungal communities across thinning intensities. The partial Mantel test was performed using the “mantel.partial” function from the *Vegan* R package to assess associations between microbial communities and environmental variables. Additionally, variance partitioning analysis (VPA) was performed using the varpart function of the *Vegan* package in R to assess the relative importance of soil carbon and nitrogen components in shaping the soil microbial community composition. Finally, we utilized the “Boruta” algorithm, a random forest-based analysis implemented in R, to identify significant factors influencing thinning intensities. All graphs were generated using the ggplot2 package in R, and Origin 2021 (OriginLab, Northampton, MA, United States), ensuring high-quality visual representations of the data.

## 3 Results

### 3.1 Soil physicochemical properties and carbon nitrogen dynamics

**Figure 2:**
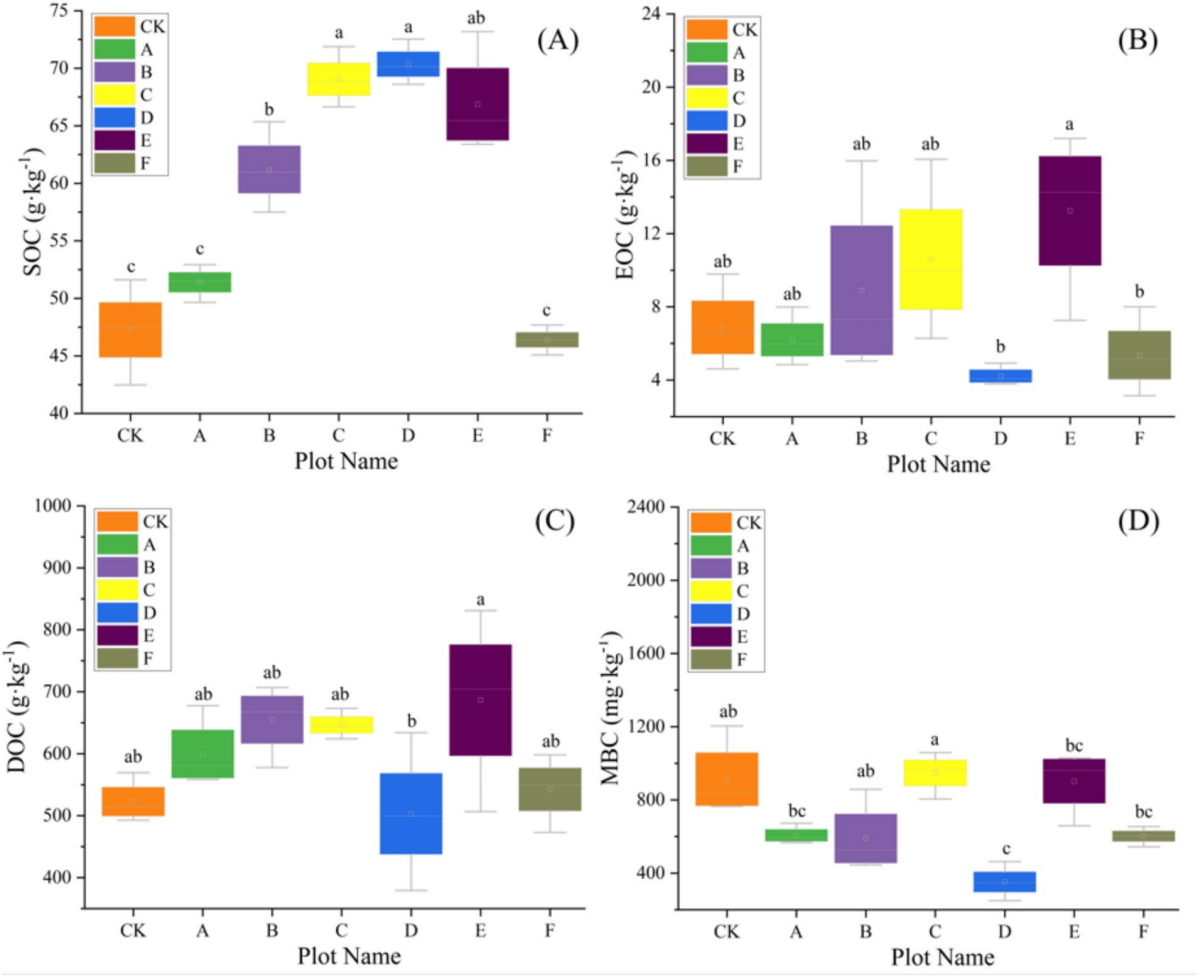
The variation of soil carbon with seven different thinning intensities (*n* = 3). **(A)** Soil organic carbon (SOC), **(B)** extractable organic carbon (EOC), **(C)** dissolved organic carbon (DOC), **(D)** microbial biomass carbon (MBC). Different letters indicate significant differences (*p* < 0.05) among the seven different thinning intensities based on a one-way ANOVA followed by an LSD test.

**Figure 3:**
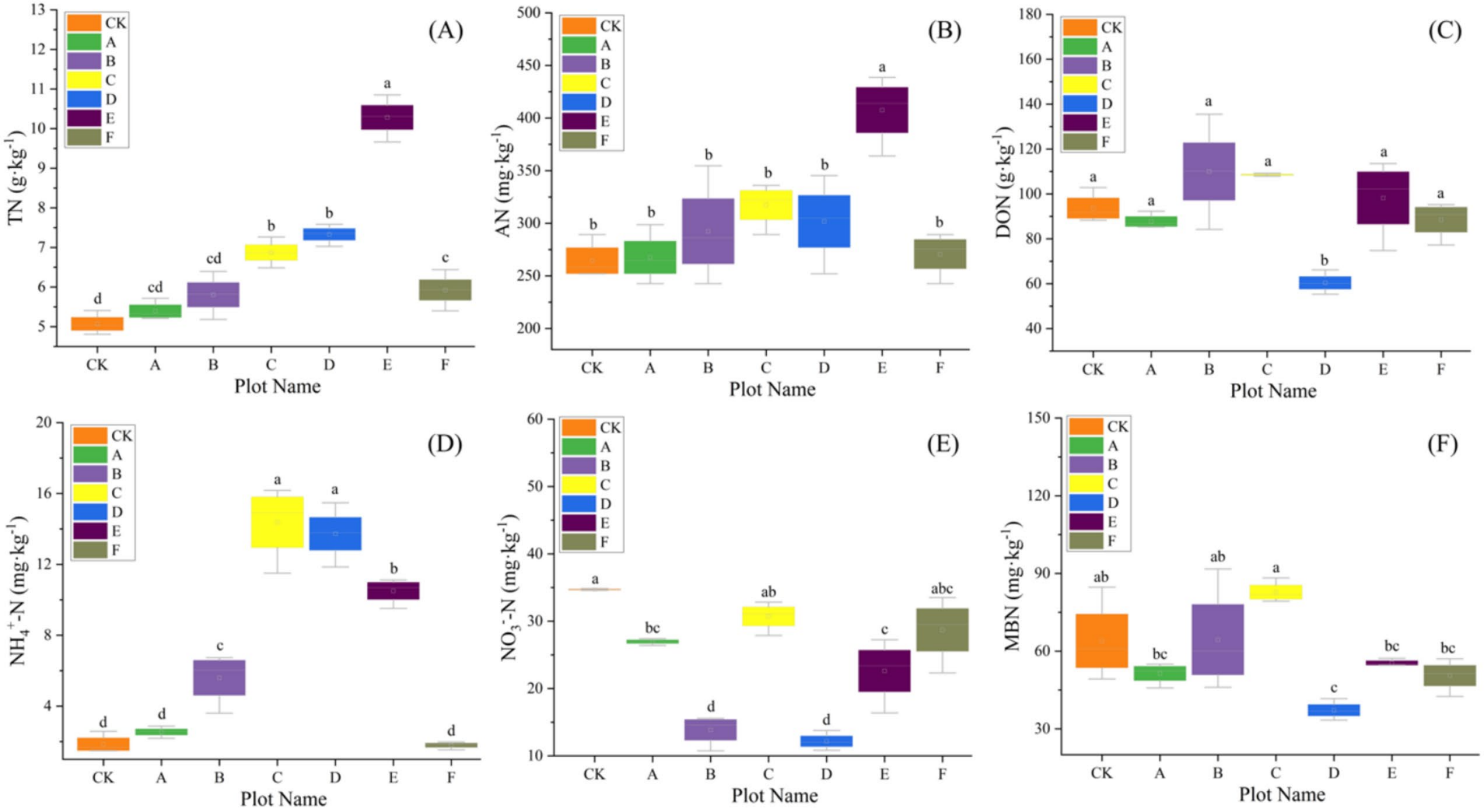
The variation of soil nitrogen with seven different thinning intensities (*n* = 3). **(A)** Total nitrogen (TN), **(B)** available nitrogen (AN), **(C)** dissolved organic nitrogen (DON), **(D)** ammonium nitrogen (NH_4_^+^-N), **(E)** nitrate nitrogen (NO_3_^−^-N), **(F)** microbial biomass nitrogen (MBN). Different letters indicate significant differences (*p* < 0.05) among the seven different thinning intensities based on a one-way ANOVA followed by an LSD test.

Baseline (pre-thinning) values for each plot are summarized in Supplementary Table S1 and serve as the reference for subsequent comparisons; across plots, SOC averaged 58.94 g kg^−1^ and TN 6.67 g kg^−1^, with bacterial α-diversity consistently high (Shannon ≈9.4, Simpson ≈0.99) and fungal diversity lower and more heterogeneous (Shannon ≈6.2). Our results revealed that thinning intensity markedly altered soil physicochemical traits and C-N dynamics, which rose at low intensities and declined at higher levels. Light thinning (10–20%) significantly elevated soil pH and concurrently increased temperature and moisture (Table 2). Heavy thinning (30–35%) reduced soil moisture and slightly lowered pH, although pH remained above the control. At moderate thinning (20–25%), TP and TK peaked at 2.50 g kg^−1^ and 11.62 g kg^−1^ (*p* < 0.001), exceeding the control by 41 and 20%, respectively. At 25% thinning (plot D), AP and AK rose to 74.04 mg kg^−1^ and 71.02 mg kg^−1^. Conversely, heavy thinning significantly lowered TP, TK, AP and AK. After controlling for elevation, slope and SI_70_, thinning intensity significantly improved model fit and remained a significant predictor for TN (ΔAIC = 3.798; *F* = 9.025, *p* = 0.020), DON (ΔAIC = 12.457; *F* = 24.950, *p* < 0.001), NH_4_^+^-N (ΔAIC = 5.807; *F* = 14.353, *p* = 0.007), MBN (ΔAIC = 8.439; *F* = 17.058, *p* < 0.001), and C/N (ΔAIC = 3.080; *F* = 7.464, *p* = 0.029). In contrast, AN (ΔAIC = 0.715; *p* = 0.111) and NO_3_^−^-N (ΔAIC = 0.451; *p* = 0.130) showed no independent thinning effect. ICC values indicated substantial between-plot heterogeneity, justifying the random-intercept structure. Full statistics are summarized in Supplementary Table S3. Light and moderate thinning raised SOC, EOC and DOC, and light thinning also elevated TN, NH_4_^+^-N and AN (*p* < 0.05; Figures 2, 3). Heavy thinning consistently suppressed soil C- and N-containing fractions. The C/N ratio peaked at 10.60 under moderate thinning but fell sharply to 6.50 with heavy thinning.

**Table 2:** Soil properties among the seven different thinning intensities.

Plot name	pH	ST (°C)	SM (%)	BD (g cm^−3^)	TP (g kg^−1^)	TK (g kg^−1^)	AP (mg kg^−1^)	AK (mg kg^−1^)	C/N
CK (0)	4.91 ± 0.02d	15.37 ± 0.21b	20.17 ± 0.55e	0.75 ± 0.06a	1.77 ± 0.06c	9.68 ± 0.45b	47.24 ± 1.34d	35.45 ± 2.71c	9.32 ± 0.74a
A (10%)	5.03 ± 0.08cd	17.63 ± 0.40a	35.87 ± 0.25b	0.66 ± 0.08a	1.75 ± 0.05c	9.74 ± 1.23b	48.49 ± 1.58d	55.64 ± 8.27ab	9.55 ± 0.57a
B (15%)	5.17 ± 0.10b	16.13 ± 0.86b	31.57 ± 3.00c	0.66 ± 0.20a	1.69 ± 0.12cd	10.35 ± 1.12ab	66.68 ± 1.68b	61.02 ± 10.57ab	10.60 ± 0.99a
C (20%)	5.11 ± 0.10bc	16.20 ± 1.23b	34.20 ± 0.46b	0.46 ± 0.04b	2.23 ± 0.15b	10.43 ± 0.54ab	65.29 ± 2.60bc	65.01 ± 12.37a	10.08 ± 0.78a
D (25%)	5.32 ± 0.09a	15.60 ± 0.92b	24.20 ± 0.46d	0.78 ± 0.03a	2.50 ± 0.06a	11.62 ± 1.74a	74.04 ± 2.73a	71.02 ± 2.59a	9.61 ± 0.61a
E (30%)	5.35 ± 0.06a	15.07 ± 0.32b	40.60 ± 0.10a	0.76 ± 0.02a	2.18 ± 0.06b	10.48 ± 0.42ab	62.65 ± 1.27c	64.22 ± 10.11a	6.50 ± 0.32c
F (35%)	5.02 ± 0.03cd	16.03 ± 0.21b	13.93 ± 0.15f	0.70 ± 0.07a	1.56 ± 0.13d	8.85 ± 0.43b	40.71 ± 1.81e	46.09 ± 6.50bc	7.87 ± 0.64b
*F*	14.658	4.223	192.912	4.403	38.558	2.372	121.595	6.508	12.524
*p*	***	*	***	*	***	ns	***	**	***

The values are shown as mean ± standard deviation. Different letters indicate significant differences (*p* < 0.05) among the four different thinning intensities based on a one-way ANOVA followed by an LSD test. ns, not significant; *p < 0.05; **p < 0.01; and ***p < 0.001. ST, soil temperature; SM, soil moisture; BD, bulk density; TP, total phosphorus; TK, total potassium; AP, available phosphorus; AK, available potassium; C/N, carbon-to-nitrogen ratio. One composite per subplot (five 0–20 cm cores, 0–20 cm depth, 5-cm auger); three composites per plot; seven plots in total (*n* = 21). Group differences were tested by one-way ANOVA with LSD *post-hoc*.

### 3.2 Soil microbial community structure and diversity

**Figure 4:**
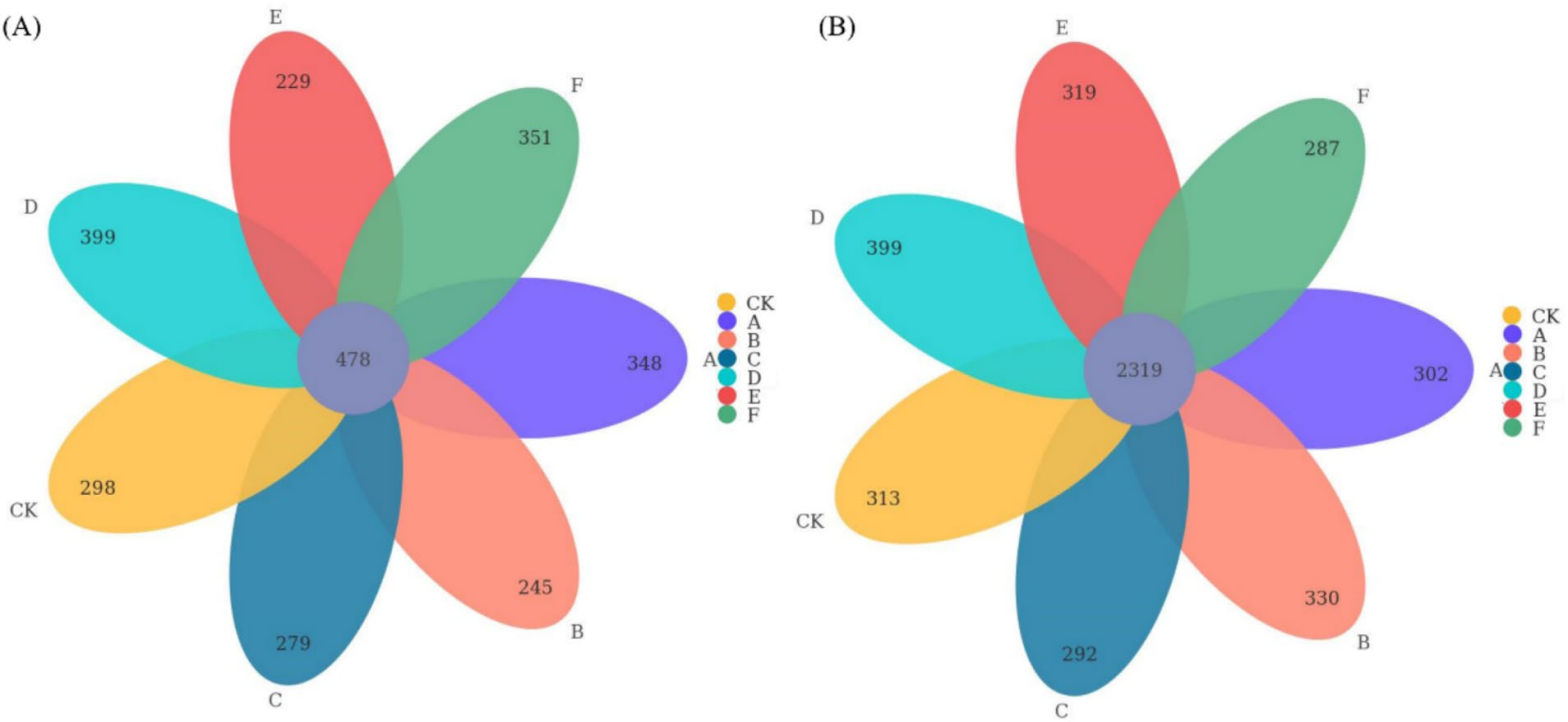
Petal plots illustrating the distribution of bacterial **(A)** and fungal **(B)** community OTUs among the seven thinning intensities.

**Figure 5:**
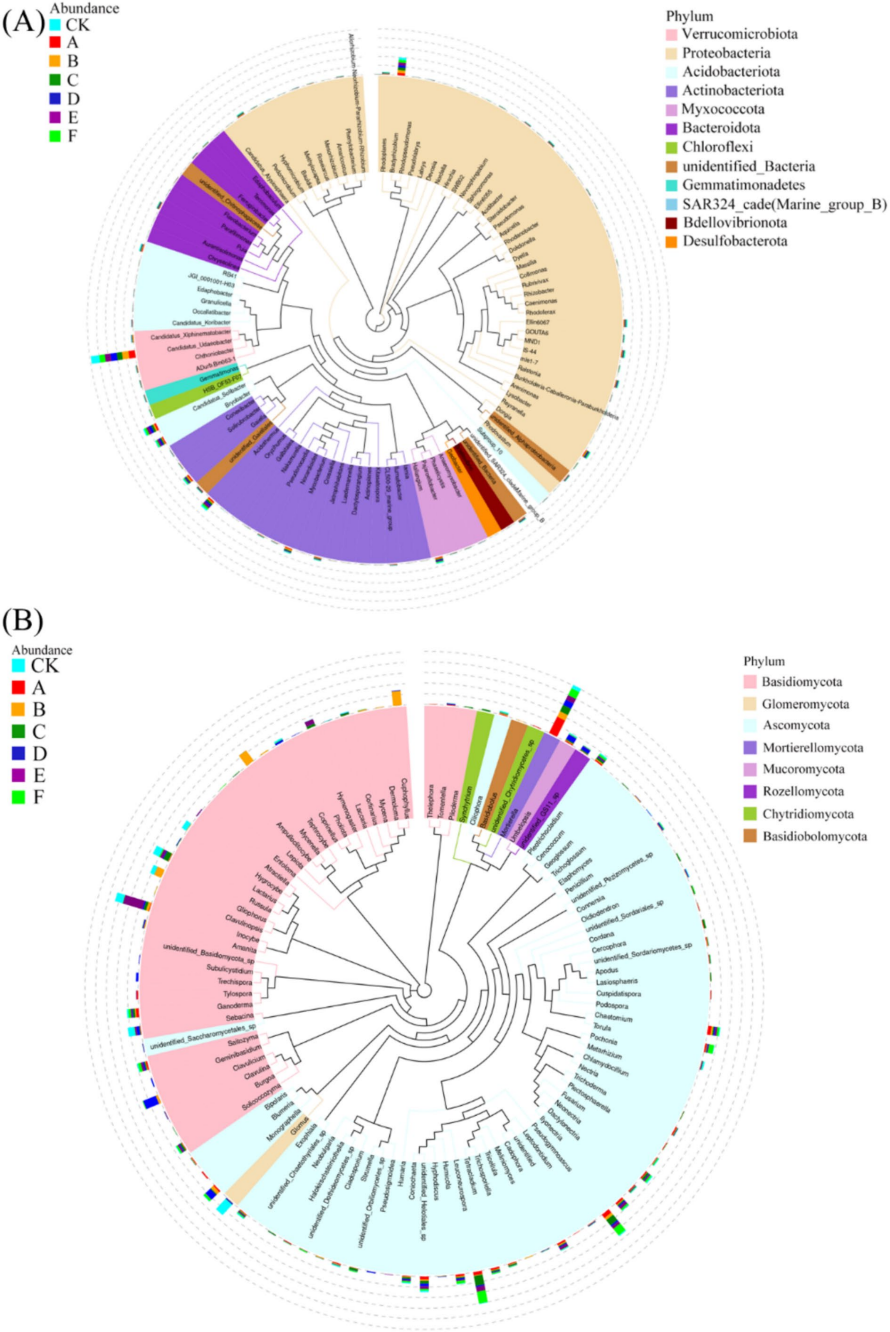
Phylogenetic trees of bacterial **(A)** and fungal **(B)** communities constructed using representative sequences at the genus level.

**Figure 6:**
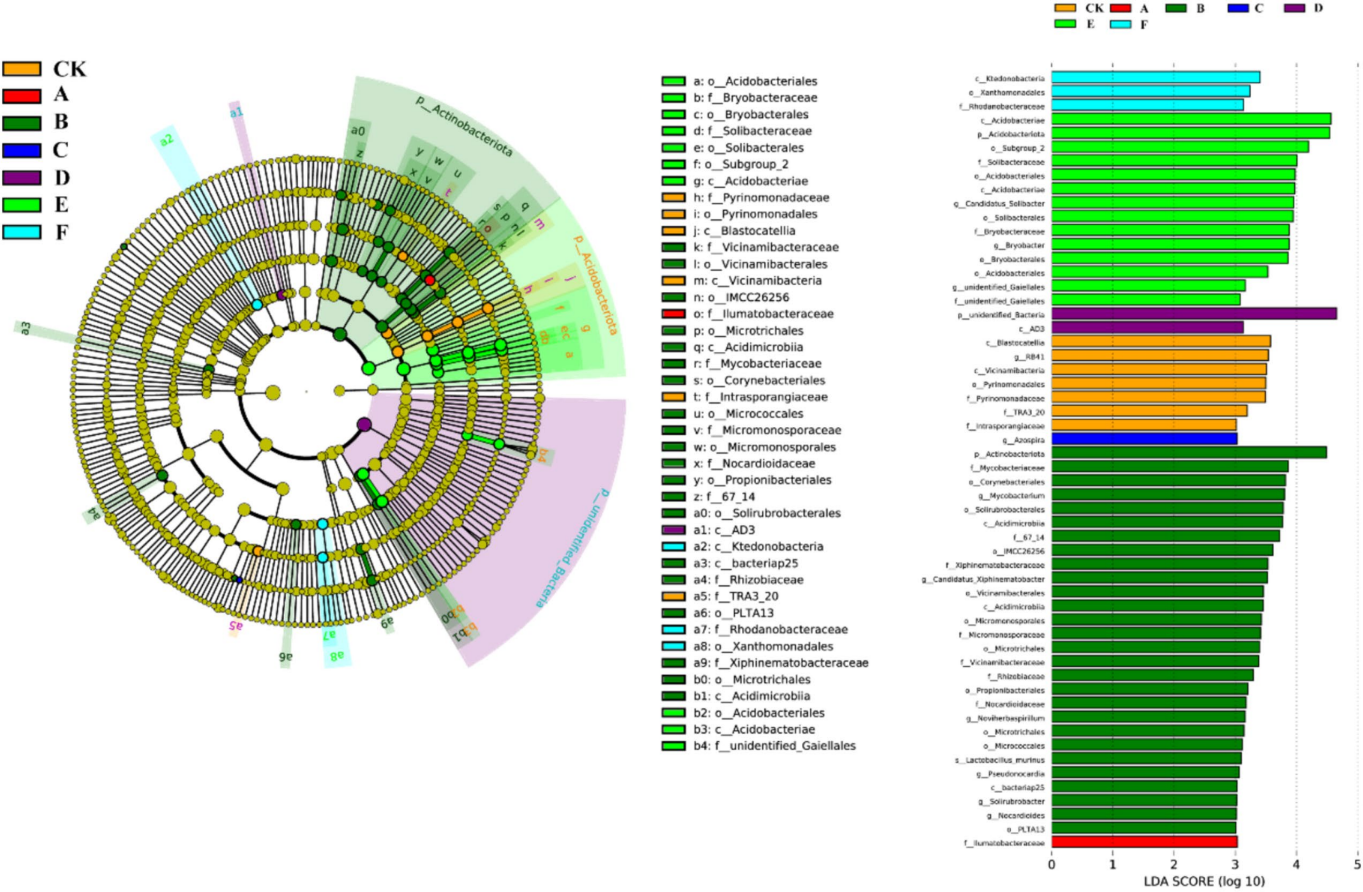
Cladogram and LDA scores of bacterial lineages in natural forest with seven thinning intensities.

**Figure 7:**
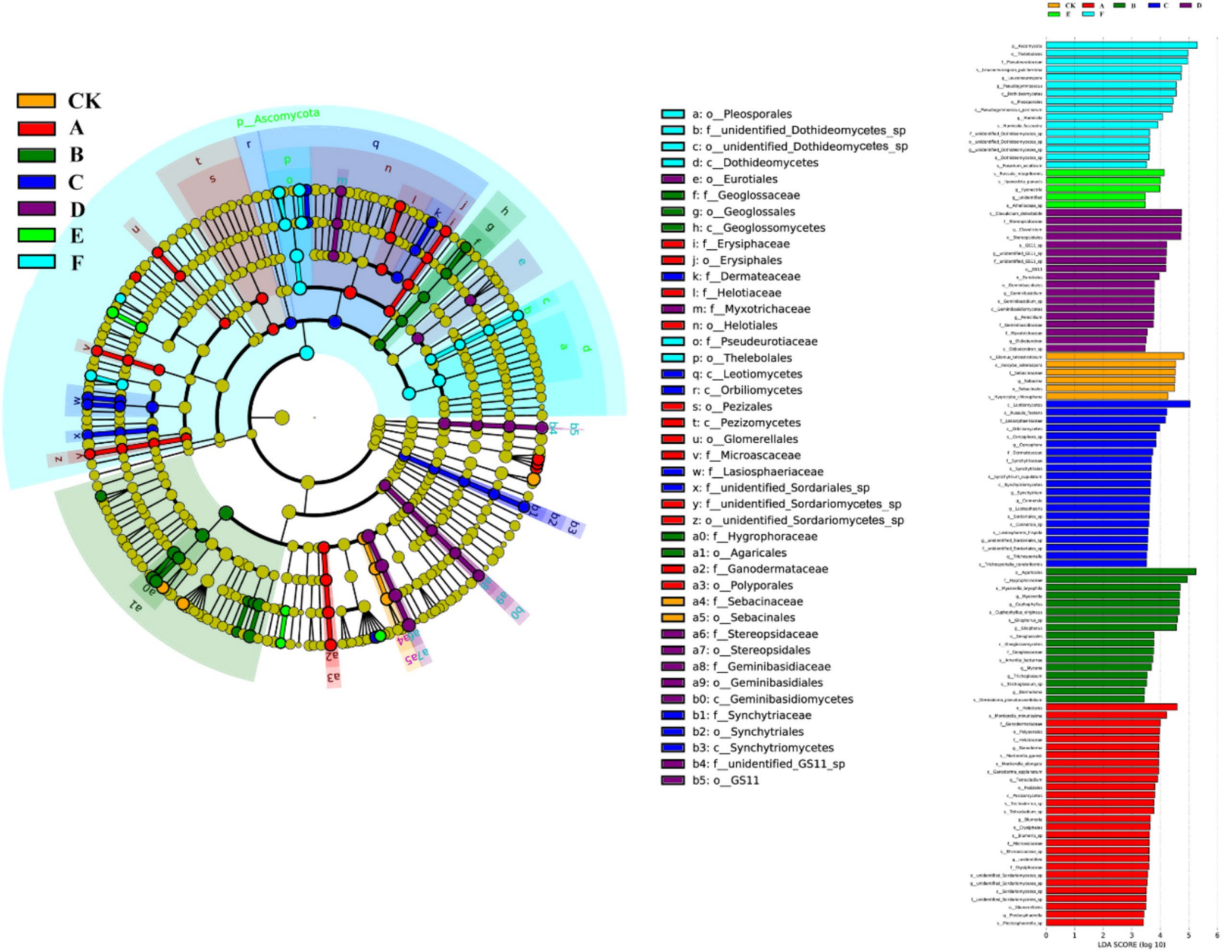
Cladogram and LDA scores of fungal lineages in natural forest with seven thinning intensities.

Thinning intensity markedly shaped soil microbial composition and functional diversity. Petal plots indicated that light thinning (10–20%) boosted diversity and unique taxa relative to the control, whereas heavy thinning (30–35%) reduced both unique and shared taxa (Figure 4). Bacteria were dominated by Verrucomicrobiota, Proteobacteria, Acidobacteriota, Actinobacteriota, Myxococcota, Bacteroidota and Chloroflexi, with Proteobacteria and Acidobacteriota most abundant (Figure 5). In light-thinning plots (A, B), Proteobacteria were enriched, whereas Bacteroidota and Chloroflexi rose under moderate-to-heavy thinning (C–F). Fungi were chiefly Basidiomycota, Ascomycota, Mortierellomycota, Mucoromycota and Glomeromycota; Basidiomycota and Ascomycota predominated. Ascomycota dominated the control (CK), whereas Basidiomycota increased under moderate-to-heavy thinning (C–F). Phylogenetic analysis revealed dense, diverse Basidiomycota lineages that expanded under heavy thinning. Phylogenetic trees and LDA (score >3.5; Figures 6, 7) corroborated the strong effect of thinning intensity on community structure. Acidobacteriales and Solibacterales prevailed at low thinning (A, B); Rhizobiales and Xanthomonadales rose at moderate thinning (C, D); Actinomycetales and drought-tolerant taxa dominated under heavy thinning (E, F). LDA showed that low-nutrient-adapted groups declined with increasing thinning, while environment-sensitive taxa peaked at moderate intensity before falling. Among fungi, Dothideomycetes and Helotiales dominated at low thinning (A, B); Sordariomycetes and Pleosporales rose at moderate thinning (C, D); drought-tolerant Agaricales and Polyporales prevailed under heavy thinning (E, F). LDA further indicated enrichment of Dothideomycetes and Sordariomycetes at low-moderate thinning, whereas stress-tolerant polyporales dominated under heavy thinning.

### 3.3 Coupling relationship between carbon nitrogen dynamics and microbial communities

**Figure 8:**
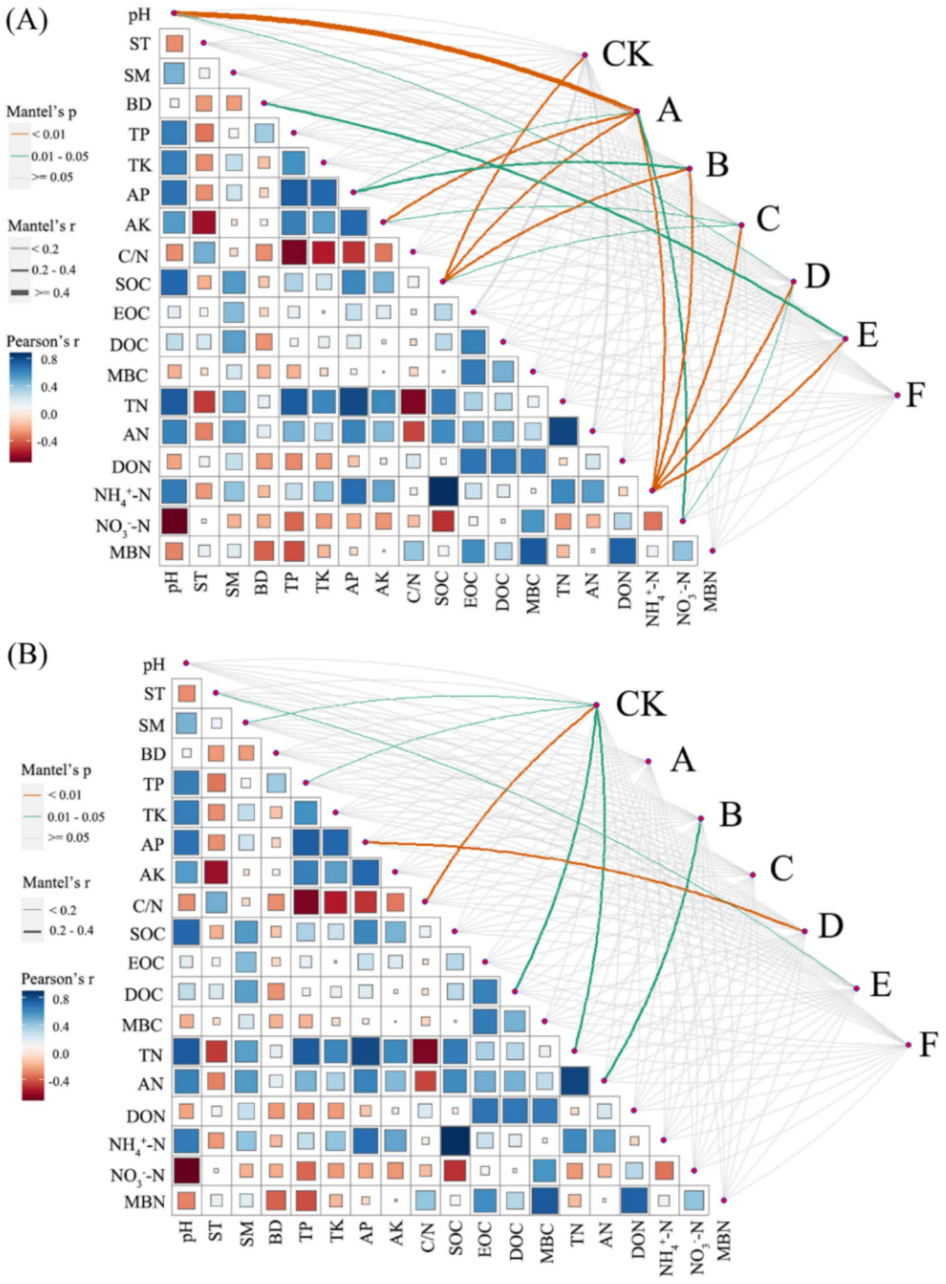
Correlations between soil properties and microbial community composition. The bacterial **(A)** and fungal **(B)** community composition, based on the Bray–Curtis distance, was examined for their relationship with each soil property using a Mantel test. The width of the lines represents the partial Mantel’s *r* statistic, while the color of the lines indicates the statistical significance based on 999 permutations. Pairwise comparisons of environmental factors were also depicted, with a color gradient representing Pearson’s correlation coefficient.

**Figure 9:**
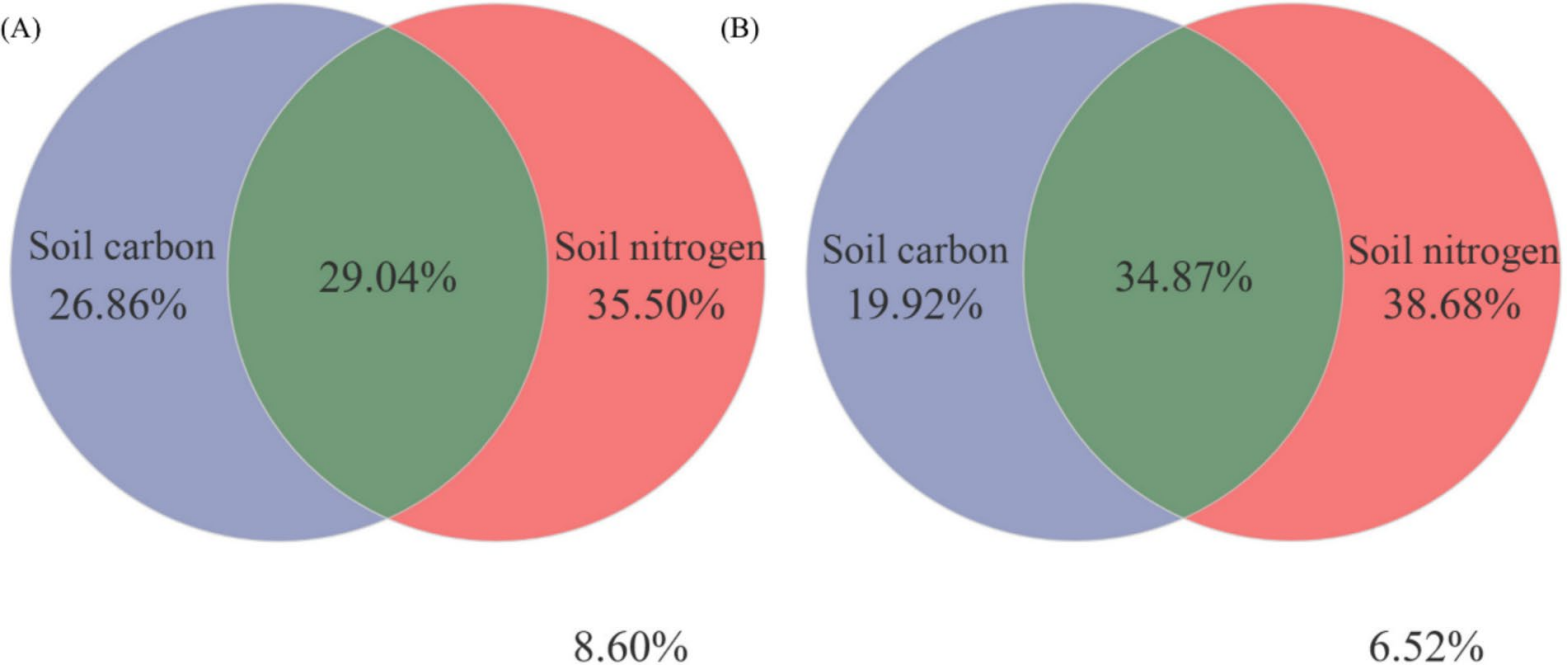
Variation partitioning analysis (VPA) was conducted to assess the contributions of soil carbon-nitrogen to the changes observed in bacterial **(A)** and fungal **(B)** communities.

**Figure 10:**
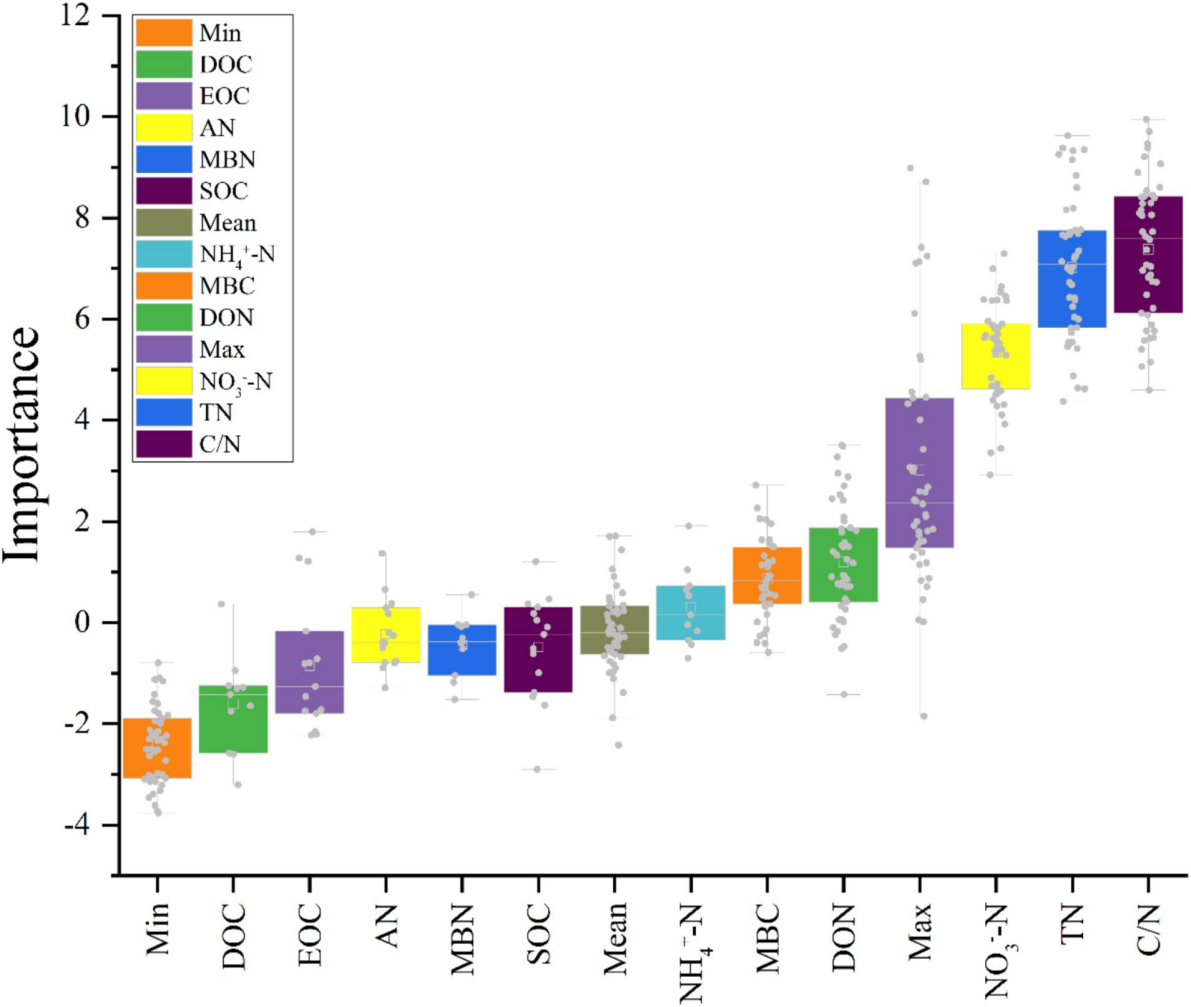
Importance of different variables over thinning intensities calculated by “Boruta.” (X-axis = soil parameters; Y-axis = importance of parameters at depth in *Z*-score).

Figure 8 illustrated, using Mantel tests and Pearson correlations, how soil physicochemical properties related to bacterial (Figure 8A) and fungal (Figure 8B) communities across thinning intensities. As thinning increased, correlations between bacterial structure and SOC, pH, and NH_4_^+^-N intensified, particularly under light to moderate thinning (10–25%), where Mantel *r* and significance (*p* < 0.01) exceeded those of heavy thinning. In contrast, fungal responses were simpler: C/N, DOC and TN correlated most strongly in CK plots, whereas AP correlation peaked under moderate thinning. Correlations between soil properties and microbial communities were weak in unthinned controls but strengthened after thinning, especially for SOC, TN and NH_4_^+^-N; the increase was larger for bacteria than fungi. Figure 9 (Venn diagrams) partitioned the variance in bacterial (Figure 9A) and fungal (Figure 9B) communities into unique and shared effects of carbon (SOC, EOC, DOC, MBC) and nitrogen (TN, DON, NH_4_^+^-N, MBN) components. For bacteria, carbon alone explained 26.86%, nitrogen alone 35.50%, and their interaction 29.04%, highlighting nitrogen’s dominant role. For fungi, carbon explained 19.92%, nitrogen 38.68%, and their interaction 34.87%, indicating greater sensitivity to nitrogen and its synergy with carbon. Figure 10 ranked soil variables by importance under the different thinning intensities. C/N ratio (7.38), TN (7.02) and NO_3_^−^-N (5.34) provided the greatest explanatory power, underscoring the sensitivity of nitrogen dynamics to thinning. DON (1.20), MBC (0.87) and NH_4_^+^-N (0.30) were less responsive but still more important than the remaining variables.

## 4 Discussion

Forest soils act as major reservoirs in the global carbon-nitrogen cycle and harbour some of the planet’s greatest microbial diversity (Kristy et al., 2025; Zhou Q. et al., 2025). Across a 0–35% thinning gradient, we found a non-linear response of soil microbial diversity, with moderate thinning yielding the most favorable outcomes for soil properties and community structure, while heavy thinning weakened C-N pools and simplified communities.

### 4.1 Effects of thinning on soil microbial community structure

Across the gradient, thinning intensity altered microbial community diversity and composition. Moderate thinning (20–25%) elevated soil pH, moisture, and macronutrient concentrations and markedly enhanced microbial diversity, especially of functional taxa such as Acidobacteriales and Rhizobiales. In contrast, thinning intensities >30% reduced soil nutrient levels, disrupted community structure, and depleted sensitive bacterial phyla. Thus, microbial diversity followed a non-linear “rise-then-fall” pattern, suggesting that moderate thinning best preserves soil health. Interpreting these treatment responses against the pre-thinning baseline (Supplementary Table S1)-i.e., uniformly high bacterial but lower and more variable fungal diversity-helps explain why bacterial metrics changed only modestly whereas fungal metrics showed stronger context dependence. Moderate canopy opening can raise understory light while only modestly increasing diurnal temperature and maintaining soil moisture, which together promote litter decomposition, root turnover, and the production of labile substrates (e.g., DOC/EOC) (Lin et al., 2015). The resulting lower C/N and higher NH_4_^+^ availability are consistent with the positive associations we observed for diversity with SOC/DOC/TN under 10–25% thinning. By contrast, heavy canopy removal (>30%) amplifies temperature and vapor-pressure-deficit swings, accelerates surface drying, and can reduce fresh litter inputs and fine-root exudation, shifting substrates toward more recalcitrant pools and selecting for stress-tolerant taxa-patterns aligned with the declines in diversity and C-N pools we detected (Castanha et al., 2018; Clinton, 2003).

These findings accord with earlier reports: moderate thinning increases microbial diversity and facilitates ecological restoration (Zhang M. et al., 2024). Light-to-moderate thinning improves aeration and moisture, creating favorable conditions that boost microbial diversity and functional richness, and slightly elevates pH, further enhancing the chemical environment for microbial activity (Ding and Yu, 2024; Wu et al., 2025). Contrary to reports of a monotonic diversity-disturbance relationship, our high-throughput data showed that diversity gains under light or heavy thinning were less pronounced than under moderate thinning (Gao et al., 2025). We propose that severe thinning excessively opens the canopy and reduces understory cover, diminishing moisture retention and organic inputs, which in turn degrades microbial communities (Bai et al., 2025; Koutika, 2025). Although excessive thinning generally lowers diversity, we observed a compensatory increase in resilient groups such as Actinobacteria, likely because drought stress and nutrient imbalances under heavy thinning passively enrich these taxa (Yang et al., 2024). Hence, thinning effects are context dependent, reflecting site conditions and microbial adaptability rather than a uniformly negative pressure (Frey et al., 2013; Wu et al., 2019; Zhang X. et al., 2023).

High-throughput sequencing allowed us to track rare or uncultured taxa, revealing differential responses of key functional groups across thinning intensities beyond the resolution of culture-based studies (Springer et al., 2024). The observed increase in Actinobacteria under heavy thinning likely reflects their tolerance to drying and capacity to depolymerize recalcitrant carbon (lignin-rich litter, aged SOM) (Bouskill et al., 2016; DeAngelis et al., 2015). Ecologically, this shift can stabilize decomposition when labile C is scarce, but it may re-weight nutrient cycling-favoring slow C turnover and microbial N immobilization while reducing community evenness and potential functional redundancy (Xu et al., 2024a; Zhou H. et al., 2025). Over time, such selection could alter SOC stabilization pathways (e.g., toward microbial necromass formation) and decouple N transformations from carbon inputs, helping explain why AN/NO₃^−^-N did not show independent thinning effects after accounting for site factors (Kallenbach et al., 2015).

### 4.2 Relationships between soil microbial communities and soil carbon-nitrogen

This study investigated how thinning intensity modulates the relationship between soil microbial community structure and carbon-nitrogen (C-N) dynamics. After accounting for site factors, thinning showed independent effects on DON, MBN, NH_4_^+^-N, TN, and C/N, consistent with mechanisms involving substrate supply, root inputs, and microbial activity. In contrast, AN and NO_3_^−^-N showed no independent thinning effect after accounting for site factors, implying dominance of between-plot heterogeneity and N transformation pathways. Soil microorganisms are central to the C-N cycle: they decompose organic matter, transform C- and N-containing compounds, and regulate nutrient supply, thereby sustaining forest ecosystem health and productivity (Geng et al., 2012; Luo et al., 2020). Yet it remains unclear how thinning disturbance influences C-N dynamics through shifts in microbial communities. Under light-to-moderate thinning (10–25%), microbial diversity increased and correlated positively with SOC, DOC, and TN, indicating that moderate thinning is associated with C-N cycling and microbial substrate use. Conversely, heavy thinning (30–35%) reduced microbial diversity and depleted soil C-N pools, potentially impairing soil function and C-N cycling efficiency. Community structure was strongly associated with key C-N variables (e.g., SOC, NH_4_^+^-N), providing new empirical evidence for microbially driven C-N processes. These results align with previous studies: Matthews et al. (2023) reported that moderate thinning improves aeration and moisture, accelerating organic-matter decomposition and increasing available C and N, whereas Cai et al. (2020) highlighted the role of microbial diversity in facilitating C-N transformations-findings consistent with our SOC-TN-diversity correlations. Our work, however, advances beyond earlier studies by clarifying the microbial-C-N coupling mechanism (Lei et al., 2021; Zhu et al., 2015). High-throughput sequencing revealed shifts in dominant phyla (e.g., Proteobacteria, Acidobacteriota) across thinning levels, whereas earlier work often focused on single functional groups or culture-dependent assays. N fractions explained more variation in communities than C fractions, suggesting stronger microbial sensitivity to N. Despite these caveats, our data reinforce the central role of microbes in regulating C-N dynamics and show that moderate thinning (10–25%) enhances both diversity and C-N cycling. The findings offer managers evidence to adjust thinning regimes that balance economic returns with soil ecological functions. Appropriately scheduled thinning could sustain profits while preserving microbial diversity and C-N cycling, thereby supporting sustainable forestry (Wang et al., 2023; Zhang Z. et al., 2023). We also show that N pools strongly influence microbial dynamics and nutrient availability. Future management should integrate targeted N fertilization and litter practices to boost microbial activity and C-N efficiency. Overall, this work offers a new framework for exploring soil-microbe-C-N linkages and lays the groundwork for multidisciplinary studies at larger spatial and temporal scales.

### 4.3 Driving factors of soil microbial communities

This study identifies the drivers of soil microbial community structure across a thinning gradient, with emphasis on physicochemical traits, C-N fractions, moisture, and temperature. Soil microbial communities are highly dynamic and diverse, responding to multiple environmental controls-chiefly soil chemistry and nutrient supply-yet most studies examine single variables and overlook their synergistic effects under varying thinning regimes (Chen et al., 2015; Tian et al., 2021). We observed that thinning intensity, the dominant disturbance factor, reshaped community composition and diversity via changes in soil properties, C-N dynamics, and microclimate. Light-to-moderate thinning raised SOC, soil N, and microbial diversity, whereas heavy thinning depleted long-term diversity and triggered large-scale community turnover under intensified moisture and temperature fluctuations. Synergistic effects of soil temperature, moisture, C/N ratio, and specific C-N fractions emerged as major drivers of community variation. Our findings agree with Wu et al. (2018) and Fang et al. (2022) that moderate thinning enhances microbial diversity. We extend earlier work by using Mantel tests and multiple regression to quantify interactions among C/N ratio, soil temperature-moisture, and thinning intensity, revealing that moisture and temperature exert stronger effects under heavy thinning-a non-linear pattern absent from previous linear models (Griffiths et al., 2011; Lei et al., 2021; Wang et al., 2018). By coupling functional-group analysis with multiple environmental metrics, we uncovered community drivers that culture-based or single-gene studies missed (Kim et al., 2017; Yang et al., 2021). Ecologically, our study offers three insights: (1) thinning intensity alters communities via changes in C-N dynamics, temperature, and moisture, informing integrated management; (2) C/N ratio, soil moisture, and temperature are pivotal indicators for monitoring soil health; and (3) optimized thinning can bolster soil C-N reserves and carbon-sink capacity, aiding climate-change mitigation. In sum, our results clarify how thinning shapes soil microbial communities and offer a scientific basis for sustainable forest management, with practical and theoretical value.

### 4.4 Limitations and future directions

Our inference is constrained by (i) a single forest type and region, (ii) single-season sampling (mid-growing season) without winter/shoulder-season coverage, (iii) a ~10-year interval between thinning (2011) and assessment (2021), which integrates multiple post-treatment processes but limits temporal attribution, (iv) moderate sequencing depth, which captures dominant/abundant taxa but may under-detect rare biosphere members, (v) composite sampling, which is optimal for plot-level means but reduces within-subplot variance resolution, and (vi) the absence of plot-level reforestation tallies (handled statistically via PlotID random intercepts rather than as a fixed covariate).

Future work should combine multi-season and multi-site sampling with continuous microclimate logging and canopy metrics, add soil-enzyme activity assays (C-, N-, and P-acquiring enzymes) and microbial process rates (net N mineralization/nitrification), and apply stable-isotope tracers (e.g., ^13^C/^15^N) to quantify substrate use efficiency and microbially mediated C-N coupling under different thinning levels. Repeated-measures designs (pre-/post-thinning trajectories) and explicit litter/fine-root input monitoring would further resolve mechanism.

## 5 Conclusion

In conclusion, our study demonstrates that moderate thinning (10–25%) significantly enhances soil microbial diversity and improves carbon-nitrogen cycling by strengthening the interactions between microbial communities and key soil components (SOC, DOC, TN). These findings highlight the importance of implementing appropriate thinning intensities to maintain soil health and ecosystem functionality, supporting sustainable forest management practices that balance economic and ecological benefits. Conversely, high-intensity thinning (30–35%) reduces microbial diversity and soil carbon-nitrogen storage, negatively impacting soil ecological functions and diminishing carbon-nitrogen cycle efficiency. Additionally, nitrogen components exert a more significant influence on microbial community dynamics and soil nutrient availability than carbon components. Future research should incorporate long-term and multi-seasonal monitoring to better understand the persistent effects of thinning on soil microbial communities and ecosystem processes.

## Data Availability

The raw 16S rRNA and ITS reads are deposited in NCBI SRA, BioProject PRJNA1280304.

## Footnotes

^1^ https://www.arb-silva.de/

^2^ http://qiime.org/scripts/make_phylogeny.html
