# Supplementary material for: Moderate thinning enhances soil carbon-nitrogen cycling and microbial diversity in degraded mixed forests
Source: Front Microbiol. 2025 Oct 2;16:1652531. doi: 10.3389/fmicb.2025.1652531 (PMC12528090; doi:10.3389/fmicb.2025.1652531)
Supplement: Supplementary file 1 [file Table_1.DOCX]

**Table S1.** Baseline (pre-thinning) soil C-N fractions and microbial α-diversity by plot (CK, A-F) and across all plots (n=21).

| Variable | Unit | CK | A | B | C | D | E | F | All plots |
| --- | --- | --- | --- | --- | --- | --- | --- | --- | --- |
| SOC | g·kg^-1^ | 47.27 ± 4.59 | 51.40 ± 1.65 | 61.21 ± 3.94 | 69.05 ± 2.64 | 70.35 ± 2.00 | 66.87 ± 5.48 | 46.40 ± 1.31 | 58.94 ± 10.29 |
| EOC | mg·kg^-1^ | 6.87 ± 2.65 | 6.19 ± 1.62 | 8.90 ± 6.14 | 10.58 ± 5.00 | 4.22 ± 0.62 | 13.24 ± 5.27 | 5.36 ± 2.46 | 7.91 ± 4.44 |
| DOC | mg·kg^-1^ | 522.66 ± 41.02 | 599.52 ± 67.57 | 654.87 ± 68.20 | 646.41 ± 24.89 | 503.03 ± 127.54 | 686.39 ± 165.03 | 542.41 ± 63.85 | 593.61 ± 102.79 |
| MBC | mg·kg^-1^ | 913.06 ± 252.21 | 606.55 ± 57.31 | 588.96 ± 233.14 | 947.44 ± 129.61 | 351.69 ± 107.07 | 902.37 ± 211.11 | 601.92 ± 55.38 | 701.71 ± 254.26 |
| TN | g·kg^-1^ | 5.07 ± 0.31 | 5.39 ± 0.28 | 5.80 ± 0.61 | 6.87 ± 0.39 | 7.33 ± 0.28 | 10.28 ± 0.60 | 5.93 ± 0.52 | 6.67 ± 1.73 |
| AN | mg·kg^-1^ | 264.44 ± 21.55 | 267.56 ± 28.51 | 292.44 ± 57.03 | 317.33 ± 24.69 | 301.78 ± 46.98 | 407.56 ± 38.86 | 270.67 ± 24.69 | 303.11 ± 56.59 |
| DON | mg·kg^-1^ | 93.63 ± 8.01 | 87.73 ± 3.96 | 110.00 ± 25.65 | 108.55 ± 0.63 | 60.42 ± 5.44 | 98.20 ± 20.61 | 88.51 ± 9.81 | 92.43 ± 19.46 |
| NH4N | mg·kg^-1^ | 1.85 ± 0.64 | 2.54 ± 0.35 | 5.60 ± 1.73 | 14.38 ± 2.51 | 13.73 ± 1.82 | 10.50 ± 0.86 | 1.79 ± 0.23 | 7.20 ± 5.43 |
| NO3N | mg·kg^-1^ | 34.70 ± 0.13 | 26.97 ± 0.52 | 13.86 ± 2.70 | 30.71 ± 2.55 | 12.16 ± 1.49 | 22.60 ± 5.60 | 28.71 ± 5.76 | 24.24 ± 8.56 |
| MBN | mg·kg^-1^ | 63.93 ± 18.50 | 51.37 ± 4.91 | 64.44 ± 24.10 | 82.76 ± 4.81 | 37.19 ± 4.18 | 55.44 ± 1.53 | 50.54 ± 7.39 | 57.95 ± 17.00 |
| Bshannon | (unitless) | 9.47 ± 0.07 | 9.45 ± 0.11 | 9.51 ± 0.07 | 9.53 ± 0.16 | 9.35 ± 0.16 | 9.40 ± 0.03 | 9.39 ± 0.11 | 9.44 ± 0.11 |
| Bsimpson | (unitless) | 0.99 ± 0.00 | 0.99 ± 0.00 | 1.00 ± 0.00 | 1.00 ± 0.00 | 0.99 ± 0.00 | 0.99 ± 0.00 | 0.99 ± 0.00 | 0.99 ± 0.00 |

**Continued Table S1.** Baseline (pre-thinning) soil C-N fractions and microbial α-diversity by plot (CK, A-F) and across all plots (n=21).

| Variable | Unit | CK | A | B | C | D | E | F | All plots |
| --- | --- | --- | --- | --- | --- | --- | --- | --- | --- |
| BACE | (unitless) | 3668.98 ± 116.13 | 3649.07 ± 155.79 | 3708.94 ± 80.78 | 3714.89 ± 86.53 | 3470.29 ± 60.49 | 3746.55 ± 499.69 | 3604.61 ± 62.54 | 3651.90 ± 196.61 |
| Bchao1 | (unitless) | 4636.08 ± 145.95 | 4671.99 ± 211.34 | 4763.35 ± 45.25 | 4654.97 ± 204.44 | 4299.89 ± 99.02 | 6622.69 ± 4076.37 | 4466.71 ± 65.57 | 4873.67 ± 1493.56 |
| Fshannon | (unitless) | 6.99 ± 0.04 | 5.47 ± 0.75 | 6.75 ± 0.13 | 6.47 ± 1.16 | 5.66 ± 0.88 | 6.69 ± 0.77 | 5.37 ± 1.18 | 6.20 ± 0.94 |

Note: Values are mean ± SD. B* = bacterial metrics; F* = fungal metrics; Soil samples 0-20 cm; units are given in the table.

**Table S2.** Stand metrics before thinning (2011) and at the time of assessment (2021), with absolute and relative changes for each plot (CK, A-F). Thinning intensity is defined as the removal rate of standing volume.

| **Plot (Intensity)** | **DBH 2011 (cm)** | **Height 2011 (m)** | **Density 2011 (trees·ha^-1^)** | **DBH 2021 (cm)** | **Height 2021 (m)** | **Density 2021 (trees·ha^-1^)** | **ΔDBH (cm)** | **%ΔDBH** | **ΔHeight (m)** | **%ΔHeight** | **ΔDensity (trees·ha^-1^)** | **%ΔDensity** |
| --- | --- | --- | --- | --- | --- | --- | --- | --- | --- | --- | --- | --- |
| CK (0%) | 16.0 ± 8.9 | 15.2 ± 7.1 | 989 | 11.39 ± 5.6 | 12.18 ± 8.6 | 1456 | -4.615 | -28.8 | -3.016 | -19.9 | 467 | 47.2 |
| A (10%) | 16.4 ± 9.2 | 14.5 ± 6.7 | 956 | 14.81 ± 3.8 | 12.62 ± 7.1 | 967 | -1.594 | -9.7 | -1.876 | -12.9 | 11 | 1.2 |
| B (15%) | 14.6 ± 8.4 | 12.0 ± 4.0 | 867 | 15.64 ± 8.2 | 12.78 ± 8.5 | 1256 | 1.036 | 7.1 | 0.778 | 6.5 | 389 | 44.9 |
| C (20%) | 11.8 ± 7.0 | 10.8 ± 3.7 | 777 | 16 ± 6.1 | 11.29 ± 4.9 | 1022 | 4.199 | 35.6 | 0.486 | 4.5 | 245 | 31.5 |
| D (25%) | 18.1 ± 8.7 | 13.1 ± 4.4 | 611 | 14.73 ± 7.2 | 11.01 ± 6.1 | 1189 | -3.37 | -18.6 | -2.09 | -15.9 | 578 | 94.6 |

**Continued Table S2.** Stand metrics before thinning (2011) and at the time of assessment (2021), with absolute and relative changes for each plot (CK, A-F). Thinning intensity is defined as the removal rate of standing volume.

| **Plot (Intensity)** | **DBH 2011 (cm)** | **Height 2011 (m)** | **Density 2011 (trees·ha^-1^)** | **DBH 2021 (cm)** | **Height 2021 (m)** | **Density 2021 (trees·ha^-1^)** | **ΔDBH (cm)** | **%ΔDBH** | **ΔHeight (m)** | **%ΔHeight** | **ΔDensity (trees·ha^-1^)** | **%ΔDensity** |
| --- | --- | --- | --- | --- | --- | --- | --- | --- | --- | --- | --- | --- |
| E (30%) | 18.5 ± 9.3 | 20.4 ± 9.5 | 567 | 13.39 ± 8.8 | 12.11 ± 9.2 | 1122 | -5.112 | -27.6 | -8.288 | -40.6 | 555 | 97.9 |
| F (35%) | 17.8 ± 8.4 | 13.1 ± 5.1 | 833 | 14.52 ± 6.7 | 13.86 ± 4.6 | 1167 | -3.279 | -18.4 | 0.756 | 5.8 | 334 | 40.1 |

Note: Values are mean ± SD. Density is trees·ha⁻¹; coordinates are plot centroids; elevation is in meters. Δ denotes 2021-2011; %Δ = Δ/2011 × 100.

**Table S3.** Linear mixed-effects models (LMMs) testing thinning intensity effects on soil nitrogen responses while accounting for between-plot heterogeneity.

| **Response** | **Model** | **Fixed effects** | **AIC** | **−2LL** | **ΔAIC** | **TI F(df)** | **TI *p*** | **ICC** |
| --- | --- | --- | --- | --- | --- | --- | --- | --- |
| TN | M0 | ZElevation + ZSlope + ZSI | 59.851 | 47.851 | - | - | - | 0.43 |
|  | M1 | M0 + TI | 56.053 | 42.053 | 3.798 | 9.025 (1,7) | 0.020 | 0.75 |
| AN | M0 | ZElevation + ZSlope + ZSI | 227.845 | 215.845 | - | - | - | 0.250 |
|  | M1 | M0 + TI | 227.130 | 213.130 | 0.715 | 3.317 (1,7) | 0.111 | 0.106 |
| DON | M0 | ZElevation + ZSlope + ZSI | 187.365 | 175.365 | - | - | - | 0.324 |
|  | M1 | M0 + TI | 174.908 | 160.908 | 12.457 | 24.950 (1,21) | <0.001 | 0.00 |

**Continued Table S3.** Linear mixed-effects models (LMMs) testing thinning intensity effects on soil nitrogen responses while accounting for between-plot heterogeneity.

| **Response** | **Model** | **Fixed effects** | **AIC** | **−2LL** | **ΔAIC** | **TI F(df)** | **TI *p*** | **ICC** |
| --- | --- | --- | --- | --- | --- | --- | --- | --- |
| NH_4_^+^-N | M0 | ZElevation + ZSlope + ZSI | 103.689 | 91.689 | - | - | - | 0.792 |
|  | M1 | M0 + TI | 97.882 | 83.882 | 5.807 | 14.353 (1,7) | 0.007 | 0.506 |
| NO_3_^-^-N | M0 | ZElevation + ZSlope + ZSI | 141.461 | 129.461 | - | - | - | 0.813 |
|  | M1 | M0 + TI | 141.010 | 127.010 | 0.451 | 2.934 (1,7) | 0.130 | 0.747 |
| MBN | M0 | ZElevation + ZSlope + ZSI | 183.053 | 171.053 | - | - | - | 0.329 |
|  | M1 | M0 + TI | 174.614 | 160.614 | 8.439 | 17.058 (1,21) | <0.001 | 0.00 |
| C/N | M0 | ZElevation + ZSlope + ZSI | 66.246 | 54.246 | - | - | - | 0.52 |
|  | M1 | M0 + TI | 63.166 | 49.166 | 3.080 | 7.464 (1,7) | 0.029 | 0.261 |

Note: Stats from LMM (random intercept for Plot; covariates: ZElevation, ZSlope, ZSI_70_; ML; Satterthwaite df). M0 = ZElevation + ZSlope + ZSI70; M1 = M0 + TI. ΔAIC = AIC(M0) − AIC(M1) (ΔAIC>0 favors M1).
